# Remote Follow-up of Self-isolating Patients With COVID-19 Using a Patient Portal: Protocol for a Mixed Methods Pilot Study (Opal-COVID Study)

**DOI:** 10.2196/35760

**Published:** 2022-08-18

**Authors:** David Lessard, Kim Engler, Yuanchao Ma, Adriana Rodriguez Cruz, Serge Vicente, Opal-COVID-19 Patient Expert Committee, Nadine Kronfli, Sapha Barkati, Marie-Josée Brouillette, Joseph Cox, John Kildea, Tarek Hijal, Marie-Pascale Pomey, Susan J Bartlett, Jamil Asselah, Bertrand Lebouché

**Affiliations:** 1 Research Institute of the McGill University Health Centre Centre for Outcomes Research & Evaluation Montreal, QC Canada; 2 See Acknowledgments; 3 Department of Medicine Division of Infectious Diseases and Chronic Viral Illness Service McGill University Health Centre Montreal, QC Canada; 4 Department of Mechanical Engineering École Polytechnique de Montréal Montreal, QC Canada; 5 Department of Family Medicine Faculty of Medicine McGill University Montreal, QC Canada; 6 Department of Mathematics and Statistics Université de Montréal Montreal, QC Canada; 7 Department of Psychiatry Faculty of Medicine McGill University Montreal, QC Canada; 8 Medical Physics Unit McGill University Montreal, QC Canada; 9 Department of Radiation Oncology McGill University Health Centre Montreal, QC Canada; 10 Centre hospitalier de l’Université de Montréal Montreal, QC Canada; 11 Department of Medicine Divisions of Experimental Medicine, Clinical Epidemiology, Respiratory Medicine, and Rheumatology McGill University Montreal, QC Canada; 12 Department of Medicine Division of Medical Oncology McGill University Health Centre Montreal, QC Canada

**Keywords:** SARS-CoV-2, coronavirus, infectious disease, implementation science, Canada, patient portal, telehealth, telemedicine, app, health information technology, remote monitoring, mobile phone

## Abstract

**Background:**

People with COVID-19 are instructed to self-isolate at home. During self-isolation, they may experience anxiety and insufficient care. Patient portals can allow patients to self-monitor and remotely share their health status with health care professionals, but little data are available on their feasibility.

**Objective:**

This paper presents the protocol of the Opal-COVID Study. Its objectives are to assess the implementation of the Opal patient portal for distance monitoring of self-isolating patients with COVID-19, identify influences on the intervention’s implementation, and describe service and patient outcomes of this intervention.

**Methods:**

This mixed methods pilot study aims to recruit 50 patient participants with COVID-19 tested at the McGill University Health Centre (Montreal, Canada) for 14 days of follow-up. With access to an existing patient portal through a smartphone app, patients will complete a daily self-assessment of symptoms, vital signs, and mental health monitored by a nurse, and receive teleconsultations as needed. Study questionnaires will be administered to collect data on sociodemographic characteristics, medical background, implementation outcomes (acceptability, usability, and respondent burden), and patient satisfaction. Coordinator logbook entries will inform on feasibility outcomes, namely, on recruitment, retention, and fidelity, as well as on the frequency and nature of contacts with health care professionals. The statistical analyses for objectives 1 (*implementation outcomes*), 3 (*service outcomes*), and 4 (*patient outcomes*) will evaluate the effects of time and sociodemographic characteristics on the outcomes. For objectives 1 (*implementation outcomes*) and 4 (*patient outcome*s), the statistical analyses will also examine the attainment of predefined success thresholds. As for the qualitative analyses, for objective 2 (*influences on implementation*), semistructured qualitative interviews will be conducted with 4 groups of stakeholders (ie, patient participants, health care professionals, technology developers, and study administrators) and submitted for content analysis, guided by the Consolidated Framework for Implementation Research to help identify barriers to and facilitators of implementation. For objective 3 (*service outcomes*), reasons for contacting health care professionals through Opal will also be submitted for content analysis.

**Results:**

Between December 2020 and March 2021, a total of 51 patient participants were recruited. Qualitative interviews were conducted with 39 stakeholders from April to September 2021. Delays were experienced owing to measures taken at the McGill University Health Centre to address COVID-19. The quantitative and qualitative analyses began in May 2022. As of June 2022, a total of 2 manuscripts (on the implementation and the patient outcomes) were being prepared, and 3 conference presentations had been given on the study’s methods.

**Conclusions:**

This protocol is designed to generate multidisciplinary knowledge on the implementation of a patient portal–based COVID-19 care intervention and will lead to a comprehensive understanding of feasibility, stakeholder experience, and influences on implementation that may prove useful for scaling up similar interventions.

**Trial Registration:**

ClinicalTrials.gov NCT04978233; https://clinicaltrials.gov/ct2/show/NCT04978233

**International Registered Report Identifier (IRRID):**

DERR1-10.2196/35760

## Introduction

### Background

As of December 13, 2021, over 267 million people worldwide have had COVID-19, including almost 2 million in Canada [1]. In 2020, without effective vaccines or treatments, governments and public health agencies enforced lockdown and distancing measures to limit the spread of COVID-19 and the burden on health care systems. At the time, in the Canadian province of Québec, 95% of people diagnosed with COVID-19 were instructed to self-isolate at home and contact health care services if their health deteriorated. While most patients with COVID-19 have mild to no symptoms in the first week following infection [2,3], patient health can rapidly deteriorate in the second week and opportunistic infections can appear [4]. Quick recognition of worsening health is crucial to avoid delays in health care and improve prognosis. However, most self-isolating patients lack tools to self-monitor their illness and maintain contact with health care professionals to ensure timely care. Self-isolation for COVID-19 can also cause anxiety, especially among vulnerable, older, or low-literacy patients [5,6], owing, in part, to inadequate information [7]. Furthermore, it can contribute to numerous other problems, including substance use or addictions [8], self-harm [9], and interpersonal difficulties [10]. Interventions to help self-isolating patients monitor symptoms and vital signs, provide them with educational materials, and facilitate communication with health care professionals could increase the safety and timeliness of care, while reducing psychological distress [5-7].

Health information technologies (HITs) offer solutions in this regard, and the pandemic has propelled their use, including in the form of telehealth and education platforms [11]. Among HITs, patient portals can provide important information and facilitate interactions between self-isolating patients and health care professionals. Their use is also associated with improved patient engagement, empowerment, and satisfaction [12]. One such portal, Opal, was initially created for use at the Cedars Cancer Centre at the McGill University Health Centre (MUHC) in Montreal, Canada, where our team is based. Implemented in 2018, it was co-designed by health care professionals, patients, and HIT developers [12]. It includes a patient smartphone app and a physician desktop dashboard. With Opal, among other advantages, oncology patients can view their diagnostics and treatment plan, laboratory test results, appointment and test schedules, clinical consultation notes, and tailored educational material. Using a dashboard designed for them, health care professionals can administer patient-reported health measures and communicate with patients between consultations [13].

To support self-isolating patients with COVID-19, our team decided to configure Opal for this purpose. This involved applying an existing HIT to a novel and different context of use. Although the benefits of HITs are well-documented for the follow-up of chronic conditions [14-16] such as cancer, little is known about their transferability to acute and typically short-term conditions such as COVID-19. Studying their implementation in this context, particularly with stakeholder engagement, a recognized key to successful HIT projects [17], would generate important data.

### Aim and Objectives

Drawing on implementation science, this mixed methods pilot study will evaluate the implementation of a patient portal (Opal) to follow-up with patients recently diagnosed with COVID-19 who were instructed to self-isolate at home. The intervention centers around daily patient self-reports of symptoms, vital signs, and mental health via Opal for remote review by a hospital-based clinical monitoring team. In line with the outcome categories proposed for implementation research [18], the study objectives are as follows: (1) to quantitatively evaluate the implementation outcomes of this intervention (in terms of predefined benchmarks for acceptability, usability, response burden, feasibility [recruitment and retention], and fidelity); (2) with qualitative data, identify the barriers to and facilitators of implementation (based on semistructured interviews with 4 stakeholder groups); (3) describe service outcomes (in terms of the rates and nature of contact with the health care professionals involved); and (4) describe patient outcomes (based on the daily self-reported health data, including symptoms, use of health services beyond the intervention, and patient satisfaction with teleconsultations, if received).

## Methods

### Study Design

This single-center pilot study follows the guidance provided by the CONSORT (Consolidated Standards of Reporting Trials) statement for pilot and feasibility studies [19,20]. It uses a mixed methods embedded design, with qualitative methods introduced secondarily to extend the breadth of inquiry [21].

### Ethics Approval

Ethics approval was obtained from the MUHC Research Ethics Board on October 5, 2020 (approval number 2021-6763), and the procedures are in accordance with the Helsinki Declaration of 1975, as revised in 2000. Participants provided informed consent and could withdraw from the study at any time. To protect their identity, no identifying information appears in any manuscript or presentation based on this study.

### Setting and Participants

#### Study Site

This study will be conducted at 2 large hospitals of the MUHC, the Glen Hospital and the Montreal General Hospital, both located in Montreal, Québec, Canada. When planning this study, from May to July 2020, at the end of the first wave of the pandemic in the province [22], approximately 150 people from local communities were tested daily for COVID-19 at the MUHC, of which approximately 15% tested positive.

#### Eligibility Criteria

The participant inclusion criteria are as follows: aged ≥18 years; fluent in French or English; a positive diagnosis at the MUHC with a polymerase chain reaction test for the virus that causes COVID-19 (SARS-CoV-2) and instruction to self-isolate at home; enrollment in Québec’s provincial health or public prescription drug insurance plan; access to a smartphone, tablet, or computer; having a home internet connection; and comfortable using a health-related smartphone app or having someone close by who is. Exclusion criteria are being hospitalized, concurrent enrollment in a COVID-19 clinical trial, and having a cognitive impairment that prevents participation.

#### Recruitment and Sample

To recruit patients, the MUHC test center staff will briefly explain the study and ask about people’s interest in participating when calling to inform them of their positive SARS-CoV-2 test result. The study coordinator will then contact interested persons to schedule a videoconferencing appointment, at which time the coordinator will request to see proof of identification (eg, passport and driver’s license) and, if applicable, collect consent. To comply with COVID-19 prevention and control measures, explicit oral consent will be obtained from all study participants via videoconferencing. An impartial witness will be present to ensure that all aspects of free and informed consent are respected. If consent is provided, the coordinator will further explain study procedures and provide baseline training on Opal.

For the collection of quantitative data (objectives 1, 3, and 4) using convenience sampling, recruitment will begin in December 2020 and continue until 50 patient participants are enrolled. This sample size meets rule-of-thumb recommendations for 1-arm pilot studies that are not designed to test effects [23,24].

For the collection of qualitative interview data (objective 2) using purposive sampling [25], the 50 MUHC participants and other stakeholders (HIT developers, administrators, and health care professionals) involved in implementing Opal for the pilot study will be invited to be interviewed. After consenting at baseline, patient participants will be asked if they would like to be contacted later for the interview. For nonpatient stakeholders, the study coordinator will record the contact information. Approximately 1 month after all patient follow-up on Opal has ended, potential interviewees will be sent an email to schedule a videoconferencing interview.

We expect that the sample achieved for objective 2 will allow for adequate saturation of the qualitative analyses, as a ≥90% representation of the main study themes is attainable with 10 to 12 qualitative interviews [26].

#### Procedures

Table 1 details all study procedures related to the patient participants, including the tasks to be accomplished throughout the 14 days of their follow-up.

**Table 1:** Study procedures for patient participants in the Opal-COVID Study^a^.

Procedure					Days of follow-up								
					Before enrollment		1		2 to 14		More days, if needed		1 month after follow-up
**Preparation**													
	COVID-19 test at MUHC^b^				✓								
	Notification of positive test result by phone and survey of interest in participation				✓								
	Study coordinator call to schedule consent process				✓								
	Informed consent and eligibility screen						✓						
	Registration and installation of Opal on participant’s device						✓						
	Educational meeting on Opal’s main functions						✓						
	Delivery of pulse oximeter or thermometer						✓						
**Data collection**													
	**Daily self-assessment**												
			Severe COVID-19 symptoms and other symptoms			✓		✓		✓			
			Vital signs			✓		✓		✓			
			Mental health (5 items)					✓ (except Days 7 and 14)		✓			
			Mental health (16 items)			✓		Only Days 7 and 14		Weekly			
		**Study questionnaire**											
			Sociodemographic and medical background information			✓							
			Implementation outcomes			✓		Only Days 7 and 14		Weekly			
			Satisfaction with care (after teleconsultations)			✓		✓		✓			
			Qualitative semistructured interview									✓	

^a^Compensation*:* CAD $50 (CAD $1=US $1.3).

^b^MUHC: McGill University Health Centre.

#### Provision of Medical Devices

The coordinator will verify whether the person owns a pulse oximeter and a thermometer, which are necessary to self-monitor vital signs during the study. Those without these devices will be able to receive them free of charge. The pulse oximeter provided will be one that is cleared by the United States Food and Drug Administration. Participants will have the option of receiving the devices through a mailing company with specific guidelines for delivery to patients with COVID-19. Alternatively, they may have them picked up by someone else at the MUHC.

### The Opal-COVID Intervention

#### Registration and Training on Opal

Patients must register to use the Opal patient portal. To do so, participating patients will be guided by a research staff member who will confirm their identity and explain Opal’s functionalities. The remote registration system will send a unique verification code to the user’s smartphone with a link to the registration page where the code will be entered. This is done to ensure the security and privacy of patient information. As shown in the provided screenshot (Multimedia Appendix 1), the user must then confirm their identity with their provincial health insurance card number, fill in some basic account information for log-in and security measures, select a data access level (ie, to allow access to their medical data or not), and accept the terms of use.

#### Duration

**Figure 1:**
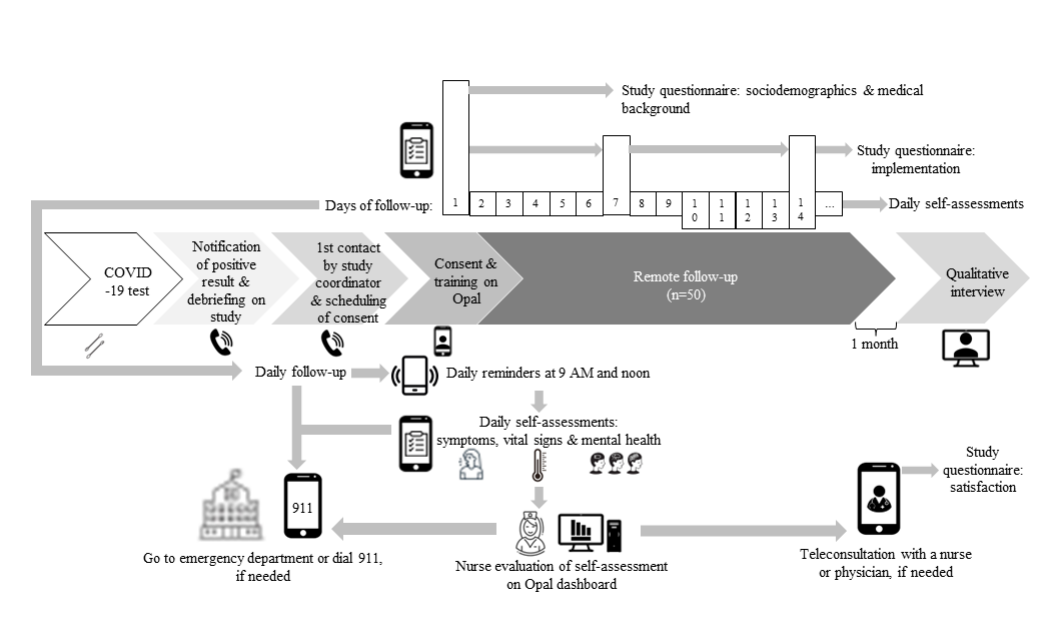
Opal-COVID Study patient participant path.

Participant follow-up is expected to last for 14 days. However, it will be prolonged in the following cases: (1) patients being diagnosed with a SARS-CoV-2 variant; (2) patients reporting fever in the last 48 hours on day 14; or (3) patients reporting other acute symptoms in the last 24 hours, except cough or loss of smell, which can take longer to subside [27]. Figure 1 shows the patient participant path in the Opal-COVID intervention.

#### Daily Self-assessments

Daily self-assessments form part of the intervention and provide most of the patient outcomes data described in objective 4. They are filled out on the Opal app in either English or French (the full self-assessment can be found in Multimedia Appendix 2; screenshots of the self-assessment are provided in Multimedia Appendix 3). From day 1 to the last day of their follow-up, these self-assessments allow patients to report symptoms, vital signs (using the pulse oximeter and thermometer), and mental health indicators to the clinical monitoring team.

The self-assessment was developed using existing COVID-19 symptom evaluation tools [27-30] and stakeholder feedback. It asks about the following: (1) the presence of severe COVID-19 symptoms (eg, severe difficulty breathing, severe chest pain, heart palpitations, feeling confused, and loss of consciousness in the past 24 hours); (2) the presence of other possible symptoms of COVID-19 (eg, pain, persistent cough, chills, runny or blocked nose, shortness of breath, loss of appetite or sense of smell or taste, sore throat, nausea or vomiting, diarrhea, abdominal cramping, unusual fatigue, and skin changes); (3) vital signs measured and reported by participants, including current temperature, respiration rate, oxygen saturation, heart rate, and, if possible, blood pressure; and (4) mental health symptoms in the past 24 hours, based on the 4-item short form versions of the Patient-Reported Outcome Measurement Information Systems (PROMIS) scales for anxiety, depression, fatigue, and sleep disturbance used under license agreement [31]. To limit response burden, participants will complete all 16 of the PROMIS items on days 1, 7, and 14, whereas on other days, they will respond to only 5.

#### Reminders

Each day, automated reminders to complete the self-assessment will be sent at 9 AM and then again at noon to participants who did not do so.

#### Monitoring

The clinical monitoring team will be composed of up to 3 nurses and 1 physician (assigned on a weekly basis).

Physicians will be available for teleconsultations conducted on the Zoom (Zoom Video Communications Inc) platform [32] and to refer patient participants to the psychiatrist (M-JB) in cases of psychological distress. All members of the clinical monitoring team will refer patient participants to emergency services as needed. The normal hours of operation for the team will be from 9 AM to 8 PM. Completed self-assessments will be reviewed by nurses on the Opal Room Management System clinical dashboard and systematically acknowledged. Each nurse will monitor up to 6 patients simultaneously. With the information provided by the self-assessment, nurses will determine a course of action, including the sending of standardized SMS text messages.

In this regard, several scenarios are possible (Multimedia Appendix 4 provides example SMS text messages sent by the nurse). For instance, if a patient’s symptoms and vital signs are stable, an SMS text message with positive feedback will be sent. If the nurse judges that a patient needs to be under closer observation, an SMS text message will be sent asking them to complete the self-assessment again. If a patient requests to speak with the nurse or is in poor condition, the nurse will book a teleconsultation for the afternoon. Opal will display the consultation time in the patient’s appointment calendar and provide a description of the consultation to help them prepare. Triggered by the nurse, an SMS text message will be sent to the patient with a link to the teleconsultation (Multimedia Appendix 5 provides screenshots relevant to teleconsultations). After each teleconsultation, to evaluate patients’ satisfaction with the care received (part of objective 4), a notification will be sent to patients through the Opal app to complete a survey based on the *Short Questionnaire for Out-of-Hours Care* [33]. It contains 7 items that are rated on a 5-point scale of satisfaction. For this study, a “not applicable” response option was added. All ratings are averaged to generate an overall score (range 1-5). Here, a mean score of ≤4 (“satisfied”) will signify adequate patient satisfaction with the teleconsultation.

If the patient completes the self-assessment after 8 PM, the clinical monitoring team will examine their responses and provide feedback early the next morning (note that the self-assessment contains instructions to go to an emergency department or call 911 if symptoms or vital signs reach specific thresholds). If no self-assessments are received from a patient for 2 consecutive days, the clinical monitoring team will reach out to them or to their emergency contact. During enrollment, these monitoring procedures are explained to participants.

### Implementation Strategies

#### Overview

This study draws on recognized implementation strategies [34] (indicated by italics). Implementation strategies are methods that are intended to foster the adoption or implementation of an intervention. The selected strategies are presented based on their corresponding main phase of implementation: preimplementation, implementation, or postimplementation.

Central to many of these strategies is our aim to *involve patients and stakeholders* through different engagement approaches. Engagement emphasizes the coconstruction of knowledge [35-38] and accountability to stakeholder values, expertise, and perspective relative to a health condition and its associated care [39]. Engagement can take many forms and encompass research and intervention development processes [40]. Importantly, it can help to detect and address challenges associated with recruitment, accessibility, acceptability, and the comprehensibility of procedures and instruments [41]. It can also increase the relevance and meaningfulness of research results [39].

Our stakeholder engagement strategy is enacted by a core multidisciplinary research team. Within this team, YM (junior engineer), BL (physician), and DL (anthropologist) were appointed to engage HIT developers, health care professionals, and patients recovered from COVID-19, respectively.

#### Strategies During the Preimplementation Phase

To *centralize technical assistance*, YM will communicate with the Opal development team (TH and JK) and maintain, configure, and update the patient portal. Regular meetings will be held to discuss the configuration of Opal for COVID-19 and ensure that it integrates stakeholder recommendations, as obtained from engaged health care professionals and patients recovering from COVID-19. Changes in Opal will be recorded via the Jira software [42]. YM will also be available to patient participants on email to provide technical assistance throughout the study.

To *create a clinical team*, BL will unite 4 physicians and 3 nurses with experience in providing COVID-19 care. Their role is to provide recommendations for the following: the use of Opal for remote COVID-19 follow-up, procedures for distance monitoring, the patient’s daily self-assessment, and information or educational material to provide to participants through the patient portal.

To engage patients with COVID-19, DL will meet with 3 people who have recovered from the disease to obtain their feedback on the proposed procedures for remote monitoring and research material (eg, informed consent documents, and all data collection instruments, including the self-assessment). This will help ensure relevance, comprehensibility, and comprehensiveness. Representation will be sought from previously infected laypersons and health care professionals with COVID-19 care experience. They will be recruited by convenience sampling within the research team’s networks.

To conduct *cyclical small tests of change*, 4 prototype tests with engaged health care professionals (ie, the clinical team) and patients are planned by YM. For each test, members attend a training on Opal, use it for 4 days, and then meet to discuss their experience, report problems, and make recommendations. Furthermore, a *low-load* run of the patient portal is planned with 5 study participants to adjust the intervention, as needed, before the “full-load” run, with all participants.

#### Strategies During the Implementation Phase

To *record user feedback*, coordinators will fill out daily field notes in a logbook. Interactions involving participants or other stakeholders, including unforeseen challenges, technical issues encountered, and any implemented solutions, will be recorded (all entry types in the coordinator logbook are provided in the Study Data Collection and Metrics section).

As described, the initial consent process with participants includes an *educational visit* to introduce Opal and its features and provide training. We will also select and create *education material*, that is, videos, frequently asked questions, and reading material, to integrate into the Opal app. This material will include COVID-19 breathing exercises [43], guidelines for self-isolation [44], pulse oximeter and thermometer user guides (Multimedia Appendix 6), and a calendar of study participant tasks. All involved stakeholder groups will review this material.

We will *promote adaptability* of the intervention and implementation by enabling adjustments to the concerns and recommendations expressed by stakeholders or participants throughout the study. Peripheral aspects of the intervention (eg, timing and content of reminders, timing of consultations, and duration of participation) are adaptable to participant needs and circumstances. For instance, every time they complete a self-assessment, participants are asked whether they wish to speak to a nurse (irrespective of their reported symptoms, vital signs, etc).

#### Strategy During the Postimplementation Phase

Our final strategy is to *purposely re-examine the implementation*. We will do this by analyzing participant and stakeholder perspectives on barriers to and facilitators of implementation after implementation (based on the qualitative interviews).

### Study Data Collection and Metrics

#### Study Questionnaires

In part to describe the sample, on day 1, participants will complete a study questionnaire that covers the following: (1) sociodemographic characteristics, including gender, age group, annual income, current occupation, postal code, ethnicity, living arrangements (eg, living alone), and job title; and (2) medical background, including smoking; other medical conditions, such as lung disease or asthma, heart disease, diabetes, kidney disease, and cancer; recent vaccination for influenza and pneumococcal disease; and use of specific medications (eg, nonsteroidal anti-inflammatory drugs, aspirin, acetaminophen, immunosuppressants, and blood pressure medication).

To assess implementation outcomes (for objective 1), a study questionnaire will be administered on a weekly basis (days 1, 7, and 14), with questions on acceptability, response burden, and usability. Acceptability indicates how agreeable, palatable, or satisfactory an intervention is to stakeholders [18]. It will be evaluated with an adapted version of the *Acceptability of Intervention Measure* [45]. The scale has 6 items rated on a 5-point Likert scale. Item scores are averaged to produce a summary score (range 1-5). In accordance with the developers’ recommendations, a minimum average score of 4 will indicate high acceptability and act as our benchmark of success.

Perceived response burden refers to the effort required to answer a questionnaire [46] and can be considered an aspect of acceptability [18]. It will be measured with a single question and a 5-point response scale that was adapted from a survey question of the United Kingdom Office for National Statistics [46]. Consistent with the previous threshold, the target will be ≥80% (41/51) of participants rating the daily self-assessments as (2) “quite easy” to (1) “very easy” to complete.

Usability refers to the “extent to which a product can be used by specified users to achieve specified goals with effectiveness, efficiency, and satisfaction” [47] and can be considered to crosscut aspects of several implementation outcomes (feasibility, appropriateness, and acceptability) [17]. It will be assessed with 3 subscales of the *Health Information Technology Usability Evaluation Scale* [48]. This questionnaire is customizable and specifically designed to evaluate mobile health technology. The selected subscales concern perceived impact (3 items), usefulness (9 items), and ease of use (5 items). Items are rated on a 5-point scale of agreement and averaged to generate subscale scores (range 1-5). A mean score of at least 4 on each subscale, indicating agreement that the technology is impactful, useful, and easy to use, will be our metric of success.

Satisfaction with teleconsultations (for objective 4) will be evaluated with a separate questionnaire sent to patients after each teleconsultation.

#### Coordinator Logbook

The coordinator logbook will capture qualitative data that will be used to assess feasibility and fidelity (for objective 1) and the rates and nature of contacts with health care professionals (for objective 3). It will include entries on the following: (1) participant questions or challenges during consent and baseline educational meetings; (2) participant recruitment, retention, and fidelity to the intervention; (3) the content of spontaneous discussions with participants or other stakeholders during implementation (eg, the object of participant calls to the coordinators); (4) all contacts with the clinical monitoring team, including clinical notes on teleconsultations with participants; (5) details on those who eventually went to an emergency department; and (6) participant compensation.

Feasibility relates to the extent to which an intervention can be successfully used in a given setting [18]. In this case, it will mainly be assessed by the following: (1) the recruitment rate, that is, the proportion of contacted eligible individuals who are included in the study and (2) the retention rate, defined as the proportion of included participants who are retained over the full follow-up period, both with a target of 75% (38/51) [49].

Fidelity, the degree to which the intervention was implemented as intended [18], will be measured as the proportion of included patients who complete their daily self-assessments over the full follow-up period, with a target of 75% (38/51).

Contacts with the clinical monitoring team will be calculated as the proportion and frequency of participants who (1) wish to talk with the nurse (based on self-assessment data); (2) receive a teleconsultation with a nurse; (3) receive a teleconsultation with a physician; (4) receive mental health support; and (5) eventually go to an emergency department for COVID-19 complications. On the basis of the details recorded in the coordinator logbook, the source of much of these data, that is, the reasons for each of these activities, will also be categorized and described.

#### Qualitative Interviews

All qualitative interviews will be conducted individually and preferentially by videoconferencing and, if not, by phone. Each interview will be recorded, last for approximately 20 minutes, and follow a semistructured guide with open-ended questions and specific prompts. Although adapted to each stakeholder group, the guide includes ≤5 broad questions on the individual’s experience of and thoughts on using Opal for COVID-19 follow-up, with prompts on challenges and facilitators [50]. Interview guides for each stakeholder group can be found in Multimedia Appendix 7.

### Data Analysis

All statistical analyses will be performed with the R software (R Foundation for Statistical Computing) [10,51].

#### Sample Description

Descriptive statistics will be used to present the participants’ sociodemographic and medical background characteristics. For continuous variables, the minimum, maximum, mean, and SD will be reported. For ordinal and nominal qualitative variables, we will report absolute and relative frequencies (proportions).

#### Objective 1: Assess Implementation Outcomes With Predefined Success Thresholds

Implementation outcomes will be summarized using descriptive statistics. Acceptability, usability, perceived response burden, and fidelity will be summarized at days 1, 7, and 14. Feasibility will be summarized at the end of the recruitment period (recruitment rate) and at the end of patient follow-up (retention rate). For continuous outcomes, the minimum, maximum, mean, and SD will be reported. For ordinal and nominal outcomes, we will report the absolute and relative frequencies (proportions). The descriptive statistics will also be stratified by gender, age group, and ethnicity.

Acceptability and usability will be evaluated using a linear mixed model (LMM) for each corresponding outcome. The dependent variable of each model will be the outcome and the independent variables will be time (days 1, 7, and 14) and 3 sociodemographic variables reported to influence patient portal use (gender [man or woman], age [≤50 years or ≥50 years], and ethnicity [White or other]) [52]. The goal of each model is to test whether the outcome’s mean score changes significantly over time and whether it differs significantly between the groups represented by the sociodemographic variables over time. LMMs are commonly used in longitudinal studies and allow participants with missing data to be retained in the analysis. Finally, if, at each time point (days 1, 7, and 14), the outcome’s mean score is greater than or equal to the predefined success threshold, we will consider the target to be met. If it is below the threshold, we will use a unilateral *t* test to test the null hypothesis of threshold attainment, since being slightly below this mark does not imply failure, given the sample mean’s variability.

The evaluation of perceived response burden and fidelity will be performed using a generalized LMM (GLMM) for each corresponding outcome, as it is appropriate when the dependent variable is not continuous. The parameters will be estimated using generalized estimating equations (GEEs), as estimates are sensitive to the specified correlation structure, particularly in noncontinuous outcomes. The independent variables will be time (days 1, 7, and 14) and the 3 sociodemographic variables. To evaluate threshold attainment, if the observed proportion is under the predefined success threshold, we will use a unilateral *z*-test, which is appropriate for hypothesis testing with proportions.

The evaluation of feasibility will be performed by confronting the observed recruitment and retention rates with the predefined success thresholds at the end of the recruitment period and patient follow-up, respectively. If the observed rates are over or equal to the success threshold, we consider that the target is met. If it is under the success threshold, we will use a unilateral *z*-test to test the null hypothesis of threshold attainment.

#### Objective 2: Identify Implementation Barriers and Facilitators

##### Overview

To identify implementation barriers and facilitators, as well as their proposed or enacted solutions, 2 trained coders will conduct a content analysis focusing on the manifest content [53] of this study’s qualitative material. This will include interview transcripts, nurses’ and physicians’ clinical notes, and coordinators’ logbook entries. Analysis will involve three phases [53]: (1) preparation, when analysts get familiar with the data set through immersion in the data; (2) organization, when they deductively code the data using the NVivo software, following a categorization matrix based on Consolidated Framework for Implementation Research (CFIR) constructs [49] to help identify barriers to and facilitators of implementation while remaining open to emerging categories; and (3) reporting, which involves presenting the analytical categories and discussing them during periodic team meetings, as well as any discrepancies in coding or interpretation, to help ensure trustworthiness [53].

##### Conceptual Framework

The CFIR will guide our qualitative analyses to categorize the barriers and facilitators identified and, quite possibly, to help interpret our findings overall. The CFIR was chosen because it includes 39 distinct constructs grouped within 5 domains of potential influence on implementation, including features of the intervention, the inner and outer settings, the individuals involved, and the implementation process [49].

#### Objective 3: Describe Service Outcomes

Service outcomes will be summarized with absolute and relative frequencies (proportions) at days 1, 7, and 14. The frequencies will also be stratified by gender, age group, and ethnicity.

Service outcome evaluation will follow the same procedure as for objective 1. As each service outcome is noncontinuous, we will use a GLMM, with parameter estimates obtained by GEE, with the same independent variables.

For objective 3, a qualitative analysis will also be performed on the reasons underlying participant contacts with the clinical monitoring team (eg, teleconsultations) and patient care-seeking in an emergency department, as textually recorded in the coordinator logbook, including the clinical monitoring team’s clinical notes. Content analysis [53], as previously described, will be used for this purpose. This will provide more context to the quantitative data on service outcomes.

#### Objective 4: Describe Patient Outcomes

The patient outcomes collected from the daily self-assessment will be summarized each day with descriptive statistics. For continuous outcomes, the minimum, maximum, mean, and SD will be reported. For ordinal and nominal qualitative outcomes, we will report absolute and relative frequencies (proportions). The descriptive statistics will also be stratified by gender, age group, and ethnicity.

The evaluation of the patient outcomes will follow the same procedure as for objective 1: an LMM for each continuous outcome and a GLMM for each noncontinuous outcome with parameter estimates obtained by GEE, using the same independent variables except for time, which will be expressed daily (day 1 to day 14). Attainment of the patient satisfaction target will also follow the procedure for objective 1, with a unilateral *t* test, when the observed mean score is below target.

#### Mixed Methods Analysis

The quantitative and qualitative data will be analyzed separately and brought together during the interpretation of results for triangulation, comparison, and improved understanding [21]. More precisely, the barriers and facilitators identified through qualitative methods (objective 2) will be used to help interpret the quantitative findings on implementation, service, and patient outcomes (objectives 1, 3, and 4).

### Confidentiality, Data Management, and Cybersecurity

The study coordinator will assign a participant number to all participants in this study to protect their identity. A digital file will contain both the participant numbers and identifying information. This file will be stored in a password-protected folder on a secure MUHC server. Interview recordings will be destroyed after transcription. Transcripts will be anonymized and stored as previously described.

Data obtained from patient participants during their follow-up (eg, sociodemographic information, daily self-assessments, and feasibility) will be electronically collected directly through Opal. The Opal team (YM, TH, JK, and JA) will oversee the management of these data, which will also be held in a secure MUHC server.

Opal conforms to security and governance recommendations for the development of patient portals, as identified by the MUHC Security and Governance team. Details are presented in Multimedia Appendix 2 of Kildea et al [12].

### Anticipated Risks and Benefits

There are no direct risks to the participants in this study. For Opal, data security risks were addressed with numerous measures, such as disclosing and explaining these to patients, logging users out after 5 minutes of inactivity, sandboxing data on the users’ device and deleting it on log-out, and including security questions in the authentication process [12]. However, patient participants may have unrealistic or unmet expectations of the intervention or a false sense of security [54] with Opal. For instance, they may overly rely on nurses to respond to the information they transmit via Opal instead of taking medical action when needed. To prevent this, the self-assessment instructs patients to seek out emergency services when specific symptoms or vital sign thresholds are attained. For various reasons (eg, ability), participants may also over- or underestimate their symptoms and vital signs, thus affecting the response they receive from the clinical monitoring team.

In addition, the study questionnaires and semistructured interviews may lead to distress related either to the experience of COVID-19 (for patients) or to providing COVID-19 care (for health care professionals). Hence, these research tasks carry a risk of emotional vulnerability. Individuals who experience psychological distress because of their involvement in the study are instructed to inform a study staff member. Resources such as a teleconsultation with a mental health professional or other support services will be made available to them.

The benefits of participating in this study for those with COVID-19 include access to the proposed intervention and follow-up. They will be compensated with CAD $50 (US $65) in the form of a gift card or, in exceptional cases, a money transfer at the end of their participation in the study.

## Results

### Status

This study secured funding from the McGill Interdisciplinary Initiative in Infection and Immunity Emergency COVID-19 Research Funding (grant ECRF-R2-44) on April 20, 2020, and from the Canadian Institutes of Health Research Strategy for Patient-Oriented Research (Quebec) Support Unit-Methodological Developments (grant M006) on February 12, 2021. Recruitment of patient participants occurred from December 2020 to March 2021, during the second wave of the pandemic in Québec, with a final sample of 51 participants [22]. Qualitative interviews were conducted between April and September 2021 during the third and fourth waves [22], with 57% (39/68) involved stakeholders, among which were 53% (27/51) patient participants, 54% (6/11) health care professionals, 100% (4/4) administrators, and 100% (2/2) HIT developers. The quantitative analyses and the qualitative content analysis of the interviews began in earnest in May 2022. The methods of this pilot study have been the object of 3 conference presentations as of June 2022 [55-57]. In addition, at this time, 2 manuscripts were being prepared, 1 involving a mixed methods analysis of the implementation (objectives 1 and 2) and the other involving patient outcomes (objective 4).

### Impact of COVID-19 on Study Progress

Early in the COVID-19 pandemic, tighter regulatory measures for data security were implemented at the MUHC. Complying with these measures delayed the acquisition of Research Ethics Board approval and study initiation. Research staff and data analysts also worked remotely to comply with social distancing measures at the MUHC, which created delays in task delegation and communication. We also experienced a shortage of trained research staff. As a result, most interviews were sent for transcription only once they had all been conducted.

## Discussion

### Anticipated Results and Contributions

We anticipate reaching the success thresholds for the studied implementation and patient outcomes. By triangulating quantitative and qualitative findings, we also expect a more complete understanding of institutional (eg, infrastructure, regulations, and human and material resources), professional (eg, expert networks), and medical (eg, symptomatology and evolution of COVID-19 infections) factors that influence the implementation of the intervention and shape its unfolding in the real world. This study will also address several gaps in the literature. Research is limited on the remote monitoring solutions for self-isolating patients with COVID-19 [58], and few such interventions have been evaluated using a mixed methods approach [59] that includes qualitative input from both patients and clinicians. Furthermore, little work has explored the configuration of HIT for chronic care management for use in acute care, such as for COVID-19 and our work will provide an example for how to proceed.

### Strengths and Limitations

Strengths of this study include its pragmatism and response to a locally identified gap in the care of people with COVID-19. Its multipronged implementation strategy, with an emphasis on stakeholder engagement and iterative feedback processes, promises to enhance intervention configuration, implementation, and evaluation. The intervention and study also benefit from the pre-existing successful use of the Opal patient portal in oncology at the study site (MUHC) [60] and collaborations between the investigators and Opal developers on other projects (eg, the studies by Chu et al [61] and Engler et al [62]). Thus, the rapid scale-up of this precise intervention at other institutions may be limited.

Limitations of this study also include several sources of potential bias. Indeed, enrollment in Québec’s provincial public health insurance plan (Régie de l'assurance-maladie du Québec) was necessary for patient inclusion, which may have introduced selection bias. Those potentially excluded include members of vulnerable populations, such as resettled refugees [63] and international students [64], groups particularly impacted by the pandemic in Canada, in part, by high COVID-19 incidence rates. In addition, for feasibility, the qualitative interviews were conducted after completing the quantitative data collection. For some participants, interviews took place several months after their follow-up ended. This time lapse may affect the reliability of the qualitative results, in part, because of recall bias. This also meant that this patient input could not be used to adjust the intervention or its implementation during enrollment and follow-up, leading to possible missed opportunities for improvement [65].

### Conclusions

Overall, this protocol is designed to generate multidisciplinary knowledge on the configuration and piloting of a patient portal–based intervention for remote COVID-19 follow-up and will lead to a comprehensive understanding of feasibility, stakeholder experience, and influences on implementation that may help in the testing or scale-up of similar interventions. It also promises to produce data that are useful for a wide range of stakeholders, including academics, researchers, health care professionals and administrators, implementation scientists, and HIT developers, who are interested in fostering feasible patient-oriented remote care for COVID-19 and beyond.

## Appendix

Multimedia Appendix 1 Screenshot of the Opal remote registration system.

Multimedia Appendix 2 Content of the daily self-assessment questionnaire.

Multimedia Appendix 3 Screenshots of the daily self-assessment questionnaire on Opal: (A) questionnaire introduction, (B) example question, and (C) answer revision before submission.

Multimedia Appendix 4 Screenshots of different standardized SMS text messages sent by nurses to patient participants, following review of their daily self-assessments: (A) positive feedback, (B) teleconsultation needed, and (C) closer monitoring needed.

Multimedia Appendix 5 Screenshots relevant to procedures involving teleconsultations: (A) teleconsultation shown in the appointment calendar, (B) appointment description to help prepare patients, and (C) teleconsultation SMS text message.

Multimedia Appendix 6 Instructional video created for participants on how to use the pulse oximeter.

Multimedia Appendix 7 Qualitative interview guides for each of the stakeholder groups.

## Abbreviations

CFIR: Consolidated Framework for Implementation Research
CONSORT: Consolidated Standards of Reporting Trials
GEE: generalized estimating equation
GLMM: generalized linear mixed model
HIT: health information technology
LMM: linear mixed model
MUHC: McGill University Health Centre
PROMIS: Patient-Reported Outcome Measurement Information Systems

## Data Availability

The data sets generated and analyzed for this study are available from the corresponding author (BL) on reasonable request.
