# Supplementary figures and images for: Remote Follow-up of Self-isolating Patients With COVID-19 Using a Patient Portal: Protocol for a Mixed Methods Pilot Study (Opal-COVID Study)
Source: JMIR Res Protoc. 2022 Aug 18;11(8):e35760. doi: 10.2196/35760 (PMC9390833; doi:10.2196/35760)

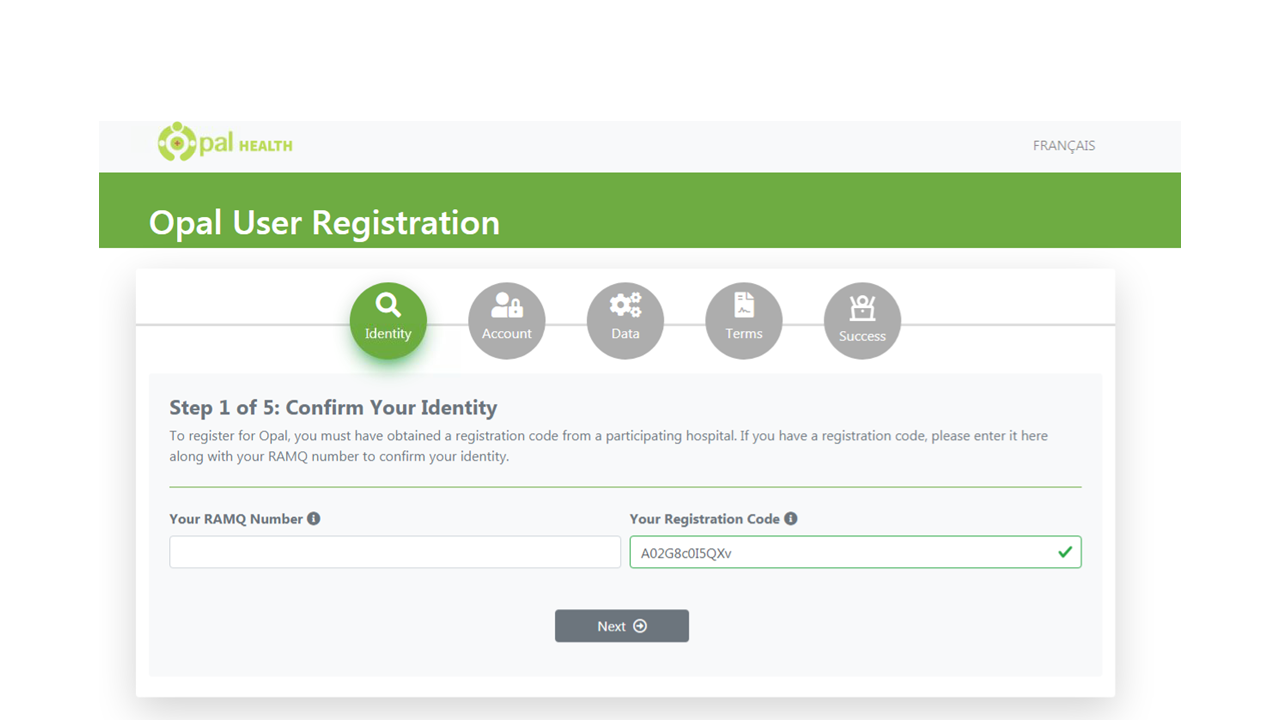

Supplement: Multimedia Appendix 1 [file resprot_v11i8e35760_app1.png]

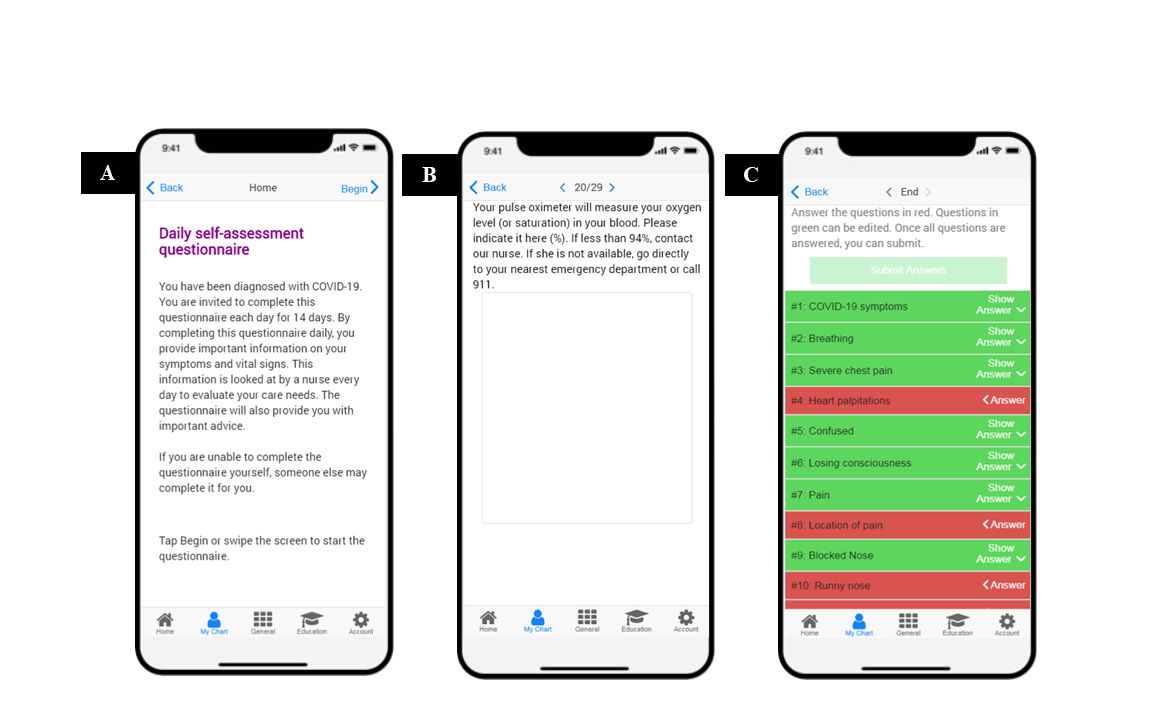

Supplement: Multimedia Appendix 3 [file resprot_v11i8e35760_app3.png]

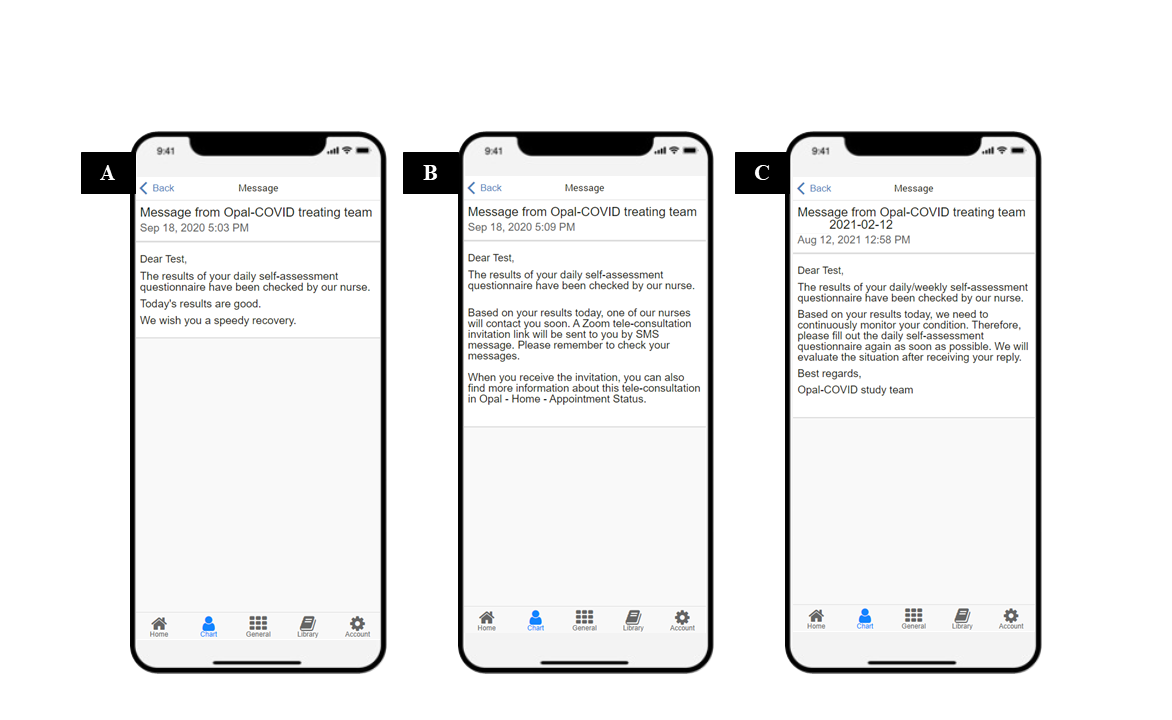

Supplement: Multimedia Appendix 4 [file resprot_v11i8e35760_app4.png]

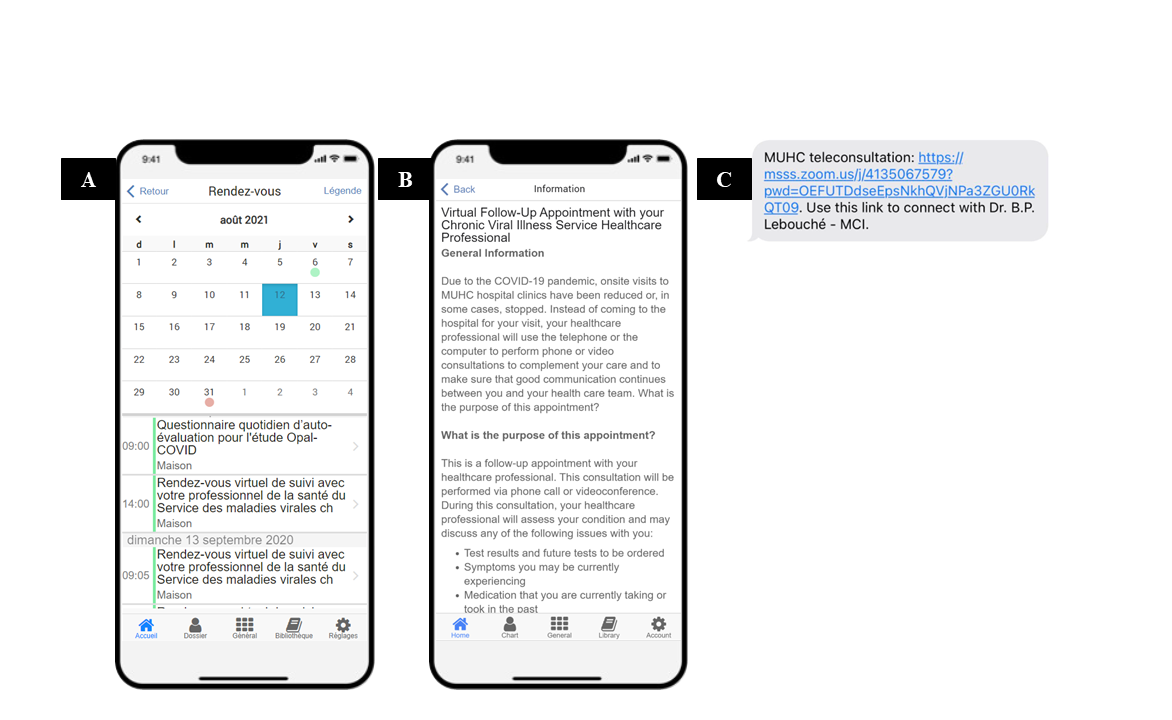

Supplement: Multimedia Appendix 5 [file resprot_v11i8e35760_app5.png]

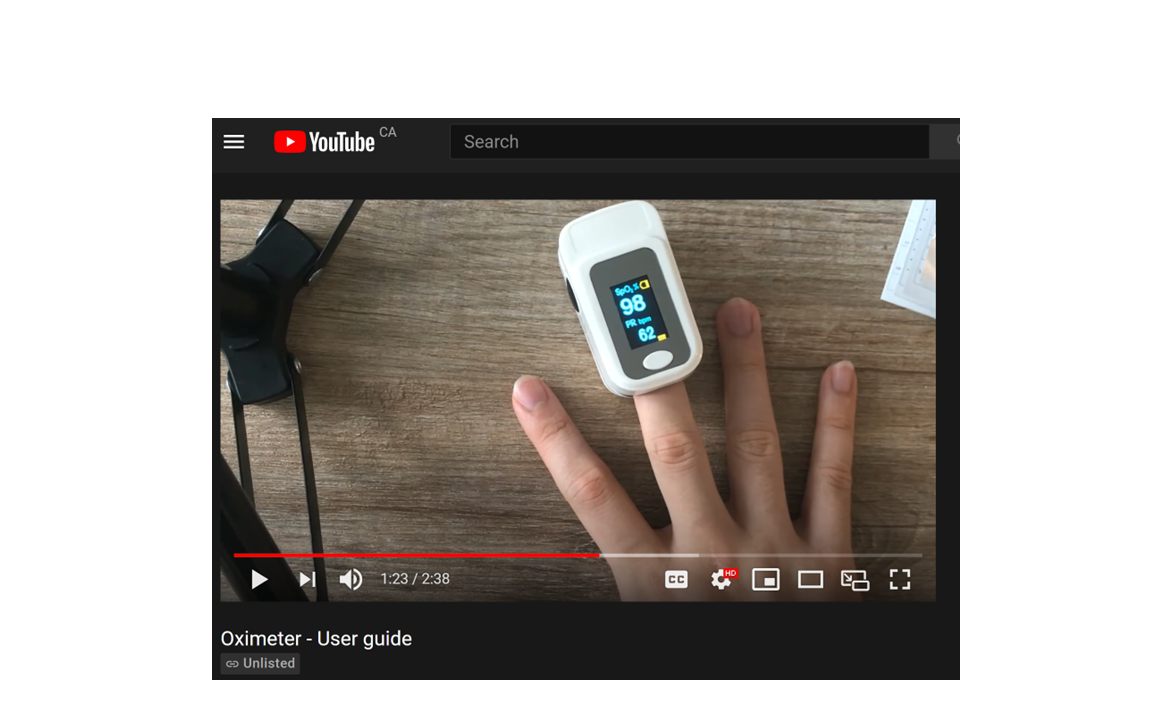

Supplement: Multimedia Appendix 6 [file resprot_v11i8e35760_app6.png]
