# Supplementary material for: Remote Follow-up of Self-isolating Patients With COVID-19 Using a Patient Portal: Protocol for a Mixed Methods Pilot Study (Opal-COVID Study)
Source: JMIR Res Protoc. 2022 Aug 18;11(8):e35760. doi: 10.2196/35760 (PMC9390833; doi:10.2196/35760)
Supplement: Multimedia Appendix 2 [file resprot_v11i8e35760_app2.docx]

**Instructions**

*You have been diagnosed with COVID-19. You are invited to complete this questionnaire each day for 14 days. By completing this questionnaire daily, you provide important information on your symptoms and vital signs. This information is looked at by a nurse every day to evaluate your care needs. The questionnaire will also provide you with important advice.*

*If you are unable to complete the questionnaire yourself, someone else may complete it for you.*

**How you feel right now**

Do you have any of the following symptoms right now?

Severe difficulty breathing (struggling for each breath, can only speak in single words)

- Yes
- No

Severe chest pain (constant tightness or a crushing sensation)

- Yes
- No

Heart palpitations (feelings of having a fast-beating, fluttering or pounding heart)

- Yes
- No

Feeling confused (for example, feeling unsure of where you are)

- Yes
- No

Have you lost consciousness or fainted in the last 24 hours?

- Yes
- No

*If you have any of these symptoms, go directly to your nearest emergency department or call 911. Mention that you are a person with COVID-19.*

Do you have pain? Choose all that apply.

- in your head (headache)
- in your muscles (myalgia, cramping)
- in your chest
- Other, please specify: __________

Is your nose blocked?

- Yes
- No

Do you have a runny nose?

- Yes
- No

Do you have a persistent cough? (coughing a lot for more than an hour, or 3 or more coughing episodes in 24 hours)

- Yes
- No

Do you have chills?

- Yes
- No

Are you experiencing unusual shortness of breath?

- Yes
- No

Have you lost your sense of smell?

- Yes
- No

*If you answered yes, do not treat it with inhaled or oral corticosteroids. This could help the virus spread. Avoid it if you can. Contact the nurse if you want more information.*

Have you lost your sense of taste?

- Yes
- No

*If you answered yes, do not treat it with inhaled or oral corticosteroids. This could help the virus spread. Avoid it if you can. Contact the nurse if you want more information.*

Do you have any of these other symptoms? Choose all that apply.

- Sore throat
- Nausea/vomiting
- Diarrhea
- Abdominal cramping
- Loss of appetite
- Unusual fatigue
- Changes to your skin

Are there other important symptoms you want to share with us? __________

**Your vital signs**

***Your current temperature***

What is your current temperature?

- I don’t have a thermometer
- Less than 38 Celsius /100.4F
- Between 38 and 39 Celsius (100.4F and 102.2F)
- More than 39 Celsius (102.2F)

*If your temperature is greater than 38 Celsius (100.4 F), and you are waiting for our nurse and/or our doctor to contact you, drink water.*

***Your respiration rate***

Check to see how many breaths per minute you are taking. Please indicate your respiration per minute here: __________per minute

*If more than 20 per minute, contact our nurse. If she is not available, go directly to your nearest emergency department or call 911.*

***Your oxygen saturation***

Your pulse oximeter will measure your oxygen level (or saturation) in your blood.
Please indicate it here: __________%

*If less than 94%, contact our nurse. If she is not available, go directly to your nearest emergency department or call 911.*

***Your heart rate***

Your pulse oximeter will measure your resting heart rate.

Please indicate here: __________beats per minute (bpm)

*If more than 100, contact our nurse. If she is not available, go directly to your nearest emergency department or call 911.*

***Your blood pressure***If you can measure your systolic blood pressure at home, please report the average of three consecutive measurements. Allow 1 min of rest between each measurement. The systolic reading is the top number.

Please indicate here: __________

*If less than 100, contact our nurse. If she is not available, go directly to your nearest emergency department or call 911.*

**Your mental wellbeing**

Please respond to each statement by marking one answer per row.

| **In the past 24 hours,…** | Never | Rarely | Sometimes | Often | Always |
| --- | --- | --- | --- | --- | --- |
| I found it hard to focus on anything other than my anxiety | ➀ | ➁ | ➂ | ➃ | ➄ |
| My worries overwhelmed me | ➀ | ➁ | ➂ | ➃ | ➄ |
| I felt helpless | ➀ | ➁ | ➂ | ➃ | ➄ |
|  | Not at all | A little bit | Somewhat | Quite a bit | Very much |
| I had trouble starting things because I am tired | ➀ | ➁ | ➂ | ➃ | ➄ |
|  | Very poor | Poor | Fair | Good | Very good |
| My sleep quality was… | ➀ | ➁ | ➂ | ➃ | ➄ |

Do you feel you should talk to the nurse today?

- Yes
- No

Indicate who completed this questionnaire. Choose one answer.

- You completed it
- Someone else completed it for you

**Below: Full mental wellbeing assessment on Days 1, 7 and 14 replaces the above section on “Your mental wellbeing”**

**Your mental wellbeing**

Please respond to each question or statement by marking one answer per row.

| **In the past 24 hours,…** | Never | Rarely | Sometimes | Often | Always |
| --- | --- | --- | --- | --- | --- |
| I felt fearful | ➀ | ➁ | ➂ | ➃ | ➄ |
| I found it hard to focus on anything other than my anxiety | ➀ | ➁ | ➂ | ➃ | ➄ |
| My worries overwhelmed me | ➀ | ➁ | ➂ | ➃ | ➄ |
| I felt uneasy | ➀ | ➁ | ➂ | ➃ | ➄ |

| **In the past 24 hours,…** | Never | Rarely | Sometimes | Often | Always |
| --- | --- | --- | --- | --- | --- |
| I felt worthless | ➀ | ➁ | ➂ | ➃ | ➄ |
| I felt helpless | ➀ | ➁ | ➂ | ➃ | ➄ |
| I felt depressed | ➀ | ➁ | ➂ | ➃ | ➄ |
| I felt hopeless | ➀ | ➁ | ➂ | ➃ | ➄ |

| **In the past 24 hours,…** | Not at all | A little bit | Somewhat | Quite a bit | Very much |
| --- | --- | --- | --- | --- | --- |
| I felt fatigued | ➀ | ➁ | ➂ | ➃ | ➄ |
| I had trouble starting things because I am tired | ➀ | ➁ | ➂ | ➃ | ➄ |
| How run-down did you feel on average? | ➀ | ➁ | ➂ | ➃ | ➄ |
| How fatigued were you on average? | ➀ | ➁ | ➂ | ➃ | ➄ |

| **In the past 24 hours,…** | Very poor | Poor | Fair | Good | Very good |
| --- | --- | --- | --- | --- | --- |
| My sleep quality was… | ➀ | ➁ | ➂ | ➃ | ➄ |
|  | Not at all | A little bit | Somewhat | Quite a bit | Very much |
| My sleep was refreshing | ➀ | ➁ | ➂ | ➃ | ➄ |
| I had a problem with my sleep | ➀ | ➁ | ➂ | ➃ | ➄ |
| I had difficulty falling asleep | ➀ | ➁ | ➂ | ➃ | ➄ |

Do you feel you should talk to the nurse today?

- Yes
- No

How did you complete this questionnaire today?

You completed it alone

Someone helped you to complete it
