# Supplementary material for: Remote Follow-up of Self-isolating Patients With COVID-19 Using a Patient Portal: Protocol for a Mixed Methods Pilot Study (Opal-COVID Study)
Source: JMIR Res Protoc. 2022 Aug 18;11(8):e35760. doi: 10.2196/35760 (PMC9390833; doi:10.2196/35760)
Supplement: Multimedia Appendix 7 [file resprot_v11i8e35760_app7.docx]

**Healthcare professionals, and technology developers and study administrators**

1) Sociodemographics

- 1. How old are you?
  2. How do you usually define your gender?
  3. What is your profesionnal title?
  4. What are your professional role and functions at the MUHC ? In the context of the Opal-COVID study? How long have you had this title and functions?
  5. How many COVID-19 patients did you follow overall? How many COVID-19 patients did you follow in the context of the Opal-COVID study?

2) Overall, describe your experience with the implementation of the Opal-COVID study, including the configuration of the app and its usage to follow COVID-19 patients self-isolating at home.

*Follow-up questions :*

- How did you get involved in the Opal-COVID study?
- What were the main steps of the preparation and implementation of the Opal-COVID study? Who were the main people involved?
- What were the main steps and the main people involved in the development of the care path associated with the Opal-COVID study?
- What were the changes brought to the Opal-COVID intervention during its implementation with COVID-19 patients self-isolating at home?

3) What do you think about the use of Opal to follow COVID-19 patients self-isolating at home?

*Follow-up questions :*

- What are the functions that are the most used? The less used?
- What do you think about the quality of care via Opal? About its security?
- What do you think about the userfriendliness of Opal?
- To which extent does Opal fulfills COVID-19 patients’ needs?
- To which extent did patients integrate Opal in their follow-up? What were the benefits for them? What were the inconveniences or risks?
- To which extent did you integrate Opal in your practice? What were the benefits for you? What were the inconveniences or risks?
- What were the impacts of Opal on your work?

*For technology developers and study administrators:*

- To which extent did healthcare professionals involved in the study integrate Opal in their practice? What were the benefits for them? What were the inconveniences or risks?
- What were the impacts for the participating patients? For healthcare professionals?
- What made the implementation of Opal-COVID easier? More difficult?

4) Overall, what are your recommendations to improve the implementation and usage of Opal or any similar platform?

**Patients**

1) Overall, describe your experience of the Opal-COVID study and follow-up.

*Follow-up questions :*

What brought you to participate in this study?

What are the main or key moments of your participation?

What particular needs or difficulties did you encounter during your participation?

What made the Opal-COVID follow-up easier? More difficult

How did you feel emotionally during this follow-up?

2) Overall, what do you think about the Opal application?

*Follow-up questions :*

- What do you think about the information presented in Opal?
- What made it difficult to use Opal?
- What made it easy to use Opal?
- How did these factors change or impact your follow-up?
- What did you like about Opal? What could be improved?
- To which extent did you feel in control during your follow-up with Opal?

3) How did the medical devices and teleconsultations, if it applies, help you during your self-isolation with COVID-19?

*Follow-up questions :*

- What was your experience of using the thermometer? The oximeter? The different questionnaires?
- What do you think about the availability of healthcare professionals involved with the Opal-COVID study?
- How did you feel after talking to the Opal-COVID research staff? The healthcare profesionnals involved in the study?

4) Overall, what are your recommendations to improve the implementation and usage of Opal or any similar platform?
